# 3G am Arbeitsplatz: zur Situation in niedersächsischen Unternehmen Ende 2021

**DOI:** 10.1007/s10273-022-3111-x

**Published:** 2022-02-19

**Authors:** Stephan L. Thomsen, Johannes Trunzer

**Affiliations:** Leibniz Universität Hannover, Institut für Wirtschaftspolitik der, Königsworther Platz 1, 30167 Hannover, Deutschland

**Keywords:** I12, I18, M54

## Abstract

Durch die Änderung des Infektionsschutzgesetzes Ende November 2021 wurde die Abbildung der 3G- und 2G-Quoten auf betrieblicher Ebene knapp ein Jahr nach Beginn der Impfungen möglich. Mithilfe einer Online-Blitzumfrage der Unternehmerverbände Niedersachsen, IHK Hannover, creditreform und dem Institut für Wirtschaftspolitik der Leibniz Universität Hannover zum Impf- und Genesenenstatus (2G), zur Zahl von Antigen-Schnelltests sowie dem Beschäftigtenanteil im Homeoffice können nun wichtige Hinweise auf die allgemeine Gefährdungslage im wirtschaftlichen und beruflichen Alltag sowie auf den damit verbundenen organisatorischen Aufwand in den Betrieben gegeben werden.

## Abstract (en)

The amendment of the Infection Protection Act at the end of November 2021 made it possible to map vaccinated, recovered and tested employees at the firm-level for the first time after the start of vaccinations in Germany. The Employers’ Associations of Lower Saxony (UVN), IHK Hannover, creditreform and the Institute for Economic Policy at Leibniz University Hanover conducted a short online survey on the vaccination and recovery status, the number of antigen rapid tests as well as the proportion of employees working from home. The results provide important indications about the general risk in everyday professional life, as well as the associated organisational effort in firms.

Am 24. November 2021 trat das neue Infektionsschutzgesetz in Kraft, das die 3G-Regelung am Arbeitsplatz sowie die Homeofficepflicht zur Verringerung des Infektionsgeschehens enthält. Aus der Pflicht zu 3G und der damit verbundenen Überprüfung ergab sich erstmals seit Beginn der Pandemie die Möglichkeit, diese Anteile auf betrieblicher Ebene abzubilden. Dies ist eine wichtige Änderung, da die bisherigen Entscheidungen über pandemiebezogene Maßnahmen im Bereich der Wirtschaft vor allem aus Annahmen sowie Übertragungen aus anderen Datenquellen (z. B. der allgemeinen Impfquote) abgeleitet wurden. Um eine wirksame Pandemiepolitik auch effizient zu gestalten, d. h. insbesondere nicht mehr Schaden insgesamt (und häufig an anderer Stelle) zu erzeugen als Nutzen zu erbringen, sind genaue(re) Kenntnisse der Betroffenheit erforderlich.

Durch die Erhebung des Impf- und Genesenenstatus (2G), ergänzt um die Zahlen von Antigen-Schnelltests sowie den Beschäftigtenanteil im Homeoffice können wichtige Hinweise auf die allgemeine Gefährdungslage im wirtschaftlichen und beruflichen Alltag sowie auf den damit verbundenen organisatorischen Aufwand in den Betrieben gegeben werden. Die Informationen auf Betriebsebene spiegeln einen wichtigen Teil der tatsächlichen Infektionsgefahren für die Beschäftigten wider, da sie einen Großteil des Tages – sofern nicht mobil arbeitend – in diesem Umfeld verbringen. Zudem können Ansatzpunkte zur Steigerung der Impfquoten aufgezeigt werden. Die nachfolgenden Ergebnisse tragen damit beispielhaft zu einer Verbesserung der Informationsbasis bei. Durch eine regelmäßige, genauere Abbildung könnten kurzfristig anstehende Entscheidungen und wirtschaftspolitische Eingriffe zielgenauer entwickelt werden. Es muss aber darauf hingewiesen werden, dass mit Blick auf das mitunter dynamische Pandemiegeschehen die Ergebnisse nur eine Momentaufnahme der Situation in Niedersachsen in der ersten Dezemberhälfte 2021 geben können. So hatte die fünfte Welle (Omikron-Variante) zum Zeitpunkt der Erhebung noch nicht begonnen.

## Datenbasis

In einer Online-Blitzumfrage vom 8. bis 14. Dezember 2021 von den Unternehmerverbänden Niedersachsen (UVN), IHK Hannover, creditreform und dem Institut für Wirtschaftspolitik der Leibniz Universität Hannover wurden Unternehmen in Niedersachsen zum Anteil geimpfter und genesener Beschäftigter, zur Zahl der täglichen Tests sowie den Anteilen im Homeoffice (zeitweise und permanent) befragt. Die Konzeption der Befragung erfolgte in Kooperation von UVN und dem Institut für Wirtschaftspolitik der Leibniz Universität Hannover. Kontaktiert wurden Unternehmen in den Verteilern des Instituts der Norddeutschen Wirtschaft (INW), der IHK Hannover und von creditreform Hannover.

**Prof. Dr. Stephan L. Thomsen** ist Direktor des Instituts für Wirtschaftspolitik und Mitglied im Vorstand des Leibniz Forschungszentrum Wissenschaft und Gesellschaft (LCSS) der Leibniz Universität Hannover.

**Johannes Trunzer** ist wissenschaftlicher Mitarbeiter am Institut für Wirtschaftspolitik der Leibniz Universität Hannover.

Die Stichprobe der antwortenden Betriebe (Stand: 14.12.2021, 10.30 Uhr) umfasst 743 Unternehmen, in denen insgesamt 87.411 Beschäftigte tätig sind. Auswertbare Fälle zu einzelnen Fragen können aufgrund fehlender Antworten abweichen. Plausibilitätschecks gegenüber offenkundig falschen Eintragungen wurden im Online-Befragungsinstrument berücksichtigt. Datenbereinigungen waren aufgrund der kurzen klaren Fragestruktur nur in wenigen Fällen erforderlich. Folgende Änderungen und Anpassungen wurden vorgenommen: Ersetzt wurde der 2G-Status (geimpfte oder genesene Beschäftigte), wenn die Angabe zum Impfstatus (geimpfte Beschäftigte) durch die Befragten maßgeblich verstanden wurde. Für die Auswertungen wurden aus den Angaben in absoluten Werten zu Beschäftigten, Geimpften oder Genesenen usw. Quotienten auf Betriebsebene berechnet, die eine Abbildung der Durchschnittsanteile über die Betriebe erlauben. Ergänzend wurden beschäftigungsgewichtete Quotienten berechnet, um die durchschnittlichen Beschäftigungsanteile ausweisen zu können.

Die Stichprobe ist eine Gelegenheitsstichprobe und nicht repräsentativ für alle Unternehmen und Beschäftigten in Niedersachsen. Daher können für die folgenden Auswertungen keine Hochrechnungen vorgenommen werden. Die Ergebnisse spiegeln aber eine grundlegende Tendenz über die 2G-Situation in niedersächsischen Betrieben sowie zu den Unterschieden zwischen Branchen und Betriebsgrößen im Dezember 2021 wider. Da die Angaben auf Betriebsebene erfolgen, ergeben die Angaben nicht direkt ein Bild der Gesamtsituation für die Beschäftigten in den Sektoren, nach Betriebsgrößen oder in den Branchen allgemein. Der Blick auf die durchschnittliche 2G-Quote im Unternehmen gibt vielmehr einen wichtigen Hinweis auf den durchschnittlichen Gefährdungsgrad am Arbeitsplatz. Im Gegensatz zu Aussagen auf Bevölkerungsebene wird so der individuellen betrieblichen Situation und damit der Alltagswirklichkeit der Beschäftigten in den Betrieben explizit Rechnung getragen.

Einige Einschränkungen durch die Wahl der Untersuchungsmethode sind aber zu beachten: Unter der Annahme, dass Beschäftigte auf eine Offenlegung verzichten und stattdessen durch regelmäßige Testungen den 3G-Status nachweisen können, stellen die berechneten 2G-Quoten eine Untergrenze des tatsächlichen 2G-Status in den Unternehmen dar. Die tatsächlichen Quoten wären in diesen Unternehmen dann höher. Daneben könnte das Antwortverhalten mit dem 2G-Status im Betrieb zusammenhängen. Betriebe mit besonders hohen oder niedrigen Impf- bzw. 2G-Quoten könnten vermehrt an der Befragung teilgenommen haben. In diesem Fall würden die tatsächlichen Quoten über- bzw. unterschätzt. Zudem beruhen alle Angaben auf Einschätzungen von Entscheider:innen in den Betrieben; die Genauigkeit kann dabei, z. B. mit zunehmender Betriebsgröße und/oder hohem Beschäftigtenanteil, der permanent mobil arbeitet, variieren.

## Empirische Ergebnisse

### Stichprobenzusammensetzung

Die Stichprobenzusammensetzung im Hinblick auf die sektorale Verteilung und die Anteile der Betriebsgrößenklassen ist in Tabelle 1 dargestellt. Bedingt durch den genutzten Adressverteiler sind Betriebe aus der Industrie im Vergleich zur tatsächlichen Situation überrepräsentiert, Handel und Dienstleistungen unterrepräsentiert. Aus demselben Grund ist auch der Anteil der Kleinstbetriebe geringer als der tatsächliche, die Anteile kleiner, mittlerer und großer Unternehmen sind entsprechend größer. Etwa 97 % der befragten Betriebe sitzen in Niedersachsen, wovon rund 40 % aus der Region Hannover stammen. Mit großem Abstand folgen Osnabrück (10 %), Celle (5 %) und Verden (4 %). Die übrigen Anteile verteilen sich relativ gleichmäßig über die Gesamtfläche des Bundeslandes.

### Geimpfte und Genesene: 2G-Quote

Tabelle 2 zeigt die Anteile der Beschäftigten, die einen Impf- oder Genesenennachweis (2G-Nachweis) vorweisen können, in Bezug zu allen Beschäftigten im Betrieb. In der ersten Spalte sind die Mittelwerte über die Anteile aller Betriebe (insgesamt sowie nach Sektoren, Betriebsgrößen und ausgewählten Branchen) dargestellt. In der zweiten Spalte sind die nach Zahl der Beschäftigten gewichteten Mittelwerte präsentiert. Während die erste Spalte also den Mittelwert über alle befragten Unternehmen zeigt und damit den durchschnittlichen Alltag bzw. die betrieblichen Kontakte wiedergibt, bleiben Größenunterschiede unberücksichtigt. Diese werden in der zweiten Spalte berücksichtigt und bilden näherungsweise die Situation für die Beschäftigten insgesamt ab. Außerdem wird der Median ausgewiesen. Er gibt an, wie hoch der Beschäftigtenanteil mit Impf- oder Genesenennachweis in der Hälfte der Betriebe ist. D. h., in der einen Hälfte der Betriebe liegt er unter dem angegebenen Anteil, in der anderen Hälfte darüber. Vervollständigt wird die Tabelle durch Angaben zur Zahl der antwortenden Unternehmen und der darin tätigen Beschäftigten. Die Tabellen für die übrigen Ergebnisse gliedern sich analog, um die Vergleichbarkeit zu verbessern.

Vor dem Hintergrund einer möglichst hohen Immunität zur Beendigung der Pandemie bzw. zur Verringerung der gesundheitlichen, wirtschaftlichen und gesellschaftlichen Schäden zeigt sich mit einem Anteil von 89,3 % im Mittel der antwortenden niedersächsischen Betriebe insgesamt eine sehr hohe 2G-Quote. Auch wenn dieser Anteil aufgrund unterschiedlicher Unternehmensgrößen nicht dem Anteil der 2G-Quote der beschäftigten Bevölkerung entspricht, ist er für wirtschaftspolitische Entscheidungen, die alle Unternehmen in gleicher Weise betreffen, relevant, weil das Ar-

Tabelle 1
**Struktur der Stichprobe**

| | Betriebe | | Beschäftigte | |
|---|---|---|---|---|
| | N | Anteil (%) | N | Anteil (%) |
| Insgesamt[1] | 743 | - | 87.411 | - |
| Nach Sektoren | | | | |
| Landwirtschaft | 7 | 1,1 | 173 | 0,2 |
| Industrie | 261 | 41,8 | 44.166 | 54,1 |
| Dienstleistungen | 357 | 57,1 | 37.264 | 45,7 |
| Nach Betriebsgrößen | | | | |
| Kleinstunternehmen | 176 | 24,5 | 981 | 1,1 |
| Kleinunternehmen | 276 | 38,4 | 7.268 | 8,3 |
| Mittlere Unternehmen | 204 | 28,4 | 24.221 | 27,7 |
| Großbetriebe | 63 | 8,8 | 54.941 | 62,9 |
| Nach ausgewählten Branchen | | | | |
| Baugewerbe | 149 | 23,8 | 15.970 | 19,6 |
| Dienstleistungen[2] | 164 | 26,2 | 16.533 | 20,3 |
| Energieversorgung | 12 | 1,9 | 13.812 | 16,9 |
| Gastgewerbe | 10 | 1,6 | 427 | 0,5 |
| Gesundheits- und Sozialwesen | 38 | 6,1 | 8.385 | 10,3 |
| Handel | 39 | 6,2 | 2.090 | 2,6 |
| Information und Kommunikation | 25 | 4,0 | 2.219 | 2,7 |
| Verarbeitendes Gewerbe | 91 | 14,6 | 13.768 | 16,9 |
| Verkehr und Lagerei | 49 | 7,8 | 4.465 | 5,5 |
| Sonstige[3] | 48 | 7,7 | 3.934 | 4,8 |

[1] bis zur letzten Seite durchgeklickt. [2] Erbringung von Finanz- und Versicherungsdienstleistungen, von freiberuflichen, wissenschaftlichen und technischen Dienstleistungen, von sonstigen Dienstleistungen und von sonstigen wirtschaftlichen Dienstleistungen. [3] alle nicht ausgewiesenen Branchen (ohne fehlende Angabe).

Anmerkung: Die Summen über die Sektoren bzw. Betriebsgrößen weichen aufgrund fehlender Angaben von „Insgesamt" ab. Alle Anteile ohne fehlende Angaben.

Tabelle 2
**2G-Quote im Dezember 2021[1]**

| | Durchschnitt | | | | |
|---|---|---|---|---|---|
| | (ungewichtet, %) | (gewichtet, %)[2] | Median (%) | N Betriebe | N Beschäftigte |
| Insgesamt | 89,3 | 83,3 | 92,9 | 693 | 83.282 |
| Nach Betriebsgrößen | | | | | |
| Kleinstunternehmen | 88,3 | 89,2 | 100,0 | 174 | 964 |
| Kleinunternehmen | 90,7 | 90,9 | 93,3 | 267 | 6.977 |
| Mittlere Unternehmen | 89,8 | 89,4 | 91,5 | 192 | 22.540 |
| Großbetriebe | 83,8 | 79,6 | 88,5 | 60 | 52.801 |
| Nach ausgewählten Branchen | | | | | |
| Baugewerbe | 86,3 | 86,2 | 89,6 | 142 | 15.066 |
| Dienstleistungen[3] | 89,7 | 76,3 | 95,5 | 157 | 16.140 |
| Energieversorgung | 81,7 | 76,7 | 91,2 | 12 | 13.812 |
| Gastgewerbe | 91,8 | 92,5 | 96,5 | 10 | 427 |
| Gesundheits- und Sozialwesen | 92,7 | 92,2 | 93,6 | 36 | 6.790 |
| Handel | 91,2 | 91,6 | 93,3 | 37 | 1.815 |
| Information und Kommunikation | 84,1 | 59,6 | 93,3 | 25 | 2.219 |
| Verarbeitendes Gewerbe | 91,3 | 88,0 | 92,9 | 88 | 13.338 |
| Verkehr und Lagerei | 89,7 | 86,9 | 92,4 | 48 | 4.413 |

[1] Frage: „Wie groß ist die Anzahl der Beschäftigten, die gegenwärtig einen Impf- oder Genesenennachweis vorweisen können?", Antworten im Verhältnis zur Beschäftigtenzahl im Betrieb. [2] nach Zahl der Beschäftigten. [3] Siehe Fußnote 2 Tabelle 1.

beitsumfeld einen wichtigen Aspekt potenzieller Kontakte darstellt. Die 2G-Quote der innerbetrieblichen Umgebung gibt einen Hinweis auf das Risiko von Ansteckungen bzw. Erkrankungen mit einem schweren Verlauf am Arbeitsplatz. Gewichtet nach Beschäftigtenzahl lag der Anteil in der ersten Dezemberhälfte bei 83,3 %. Dies ist ein Hinweis, dass die 2G-Quoten in größeren Unternehmen geringer als in kleineren sind. Hierfür sind vermutlich organisatorische Aspekte eine Erklärung, wie z. B. die höhere Anonymität der Belegschaft in Großunternehmen.[1] Die Impfquote (nicht ausgewiesen) lag nur leicht unter der 2G-Quote und zeigt in allen Differenzierungen sehr ähnliche Tendenzen. Die beschäftigungsgewichtete Impfquote lag bei 80,2 %. Sie war etwas höher als die offizielle Impfquote von 78 %, die das RKI für Niedersachsen in der Altersgruppe 18 bis 59 Jahre auswies (Stand: 15.12.2021; NDR, 2021). Die Unterschiede zwischen ungewichteter und gewichteter 2G-Quote oder auch zwischen ungewichteter und gewichteter Impfquote machen deutlich, dass differenzierte Maßnahmen speziell in größeren Unternehmen sinnvoll erscheinen.

Die Größenunterschiede bestätigen sich auch in den differenzierten Zahlen: Während die 2G-Quoten bei kleinsten bis mittleren Unternehmen zwischen 88,3 % und 90,7 % (gewichtet: 89,2 % bis 90,9 %) lagen, waren sie bei den Großbetrieben in der Stichprobe mit 83,8 % (ungewichtet) bzw. 79,6 % (gewichtet) etwas geringer. Diese Werte werden aber durch einzelne Unternehmen mit noch geringeren

1 Da Großbetriebe in der Stichprobe stark überrepräsentiert sind, würde das zugleich für eine Unterschätzung der Impfquote sprechen (alle anderen möglichen Verzerrungen einmal außer Acht gelassen).

Anteilen getrieben, wie der Vergleich mit dem Median zeigt: Die Hälfte der Großunternehmen hatte eine 2G-Quote von 88,5 % oder mehr. Gleiches gilt, wenn auch auf höherem Niveau, für die Kleinstunternehmen, bei denen die Hälfte sogar eine vollständige 2G-Quote von 100 % erreichte.

Die Betrachtung der verschiedenen Branchen zeigt keine allzu großen Unterschiede. Betriebe im Gastgewerbe, Gesundheits- und Sozialwesen, Handel und verarbeitenden Gewerbe hatten im Dezember 2021 2G-Quoten von über 90 % (ungewichtet) und in vergleichbarer Größenordnung bei Berücksichtigung der Beschäftigungsanteile. In den Sektoren Energieversorgung, Dienstleistungen und Information und Kommunikation waren die Anteile (gewichtet) jedoch deutlich geringer; etwa ein Viertel bis gut 40 % der Beschäftigten erbrachte den 2G-Nachweis nicht. Dass dies vermutlich auf eine ungleiche Verteilung zurückzuführen ist, zeigt der Blick auf den Median: Mit Ausnahme des Baugewerbes lagen die 2G-Quoten in allen Branchen bei jeweils der Hälfte der Unternehmen teilweise deutlich über 90 %.

## Testaufwand: Tests pro Kopf und Tag

Tabelle 3 gibt das Testgeschehen in den Unternehmen im Dezember 2021 wieder. Im Durchschnitt der Unternehmen wurden knapp ein Viertel der Beschäftigten pro Tag getestet (23,1 %). Die Zahlen sind nicht komplementär zum 2G-Status, weil sich auch Mitarbeiter:innen mit diesem Status testen (lassen können), weil Beschäftigte nicht in das Unternehmen kommen (mobiles Arbeiten) oder weil sich Beschäftigte außerhalb testen, z. B. durch Bürgertests.

Regelmäßige Tests sollen – insbesondere in Branchen mit hohem Kundenkontakt – das Risiko der Ansteckung der Beschäftigten sowie die Ausbreitung unter der Kundschaft verringern und beugen dadurch bedingten Produktionsausfällen oder Vertrauensverlusten vor. Sie bedeuten für die Unternehmen allerdings auch einen zusätzlichen administrativen und logistischen Aufwand und reduzieren die zur Verfügung stehende Arbeitszeit ohne Ausgleich. Hierbei unterscheiden sich die Aufwände zwischen Betrieben unterschiedlicher Größe und nach Branchen teilweise erheblich. Während bei Kleinstunternehmen knapp ein Viertel der Belegschaft (24,3 %) täglich getestet werden musste, lag der Anteil bei Großunternehmen bei etwa jedem/r zehnten Beschäftigten (10,5 %). Entsprechend ist davon auszugehen, dass auch die dadurch induzierten Produktionsausfälle bei den größeren Unternehmen geringer sind.

Deutliche Unterschiede zeigen sich auch zwischen den Branchen. Die höchste Belastung hatte das Gastgewerbe; hier wurden im Durchschnitt mehr als die Hälfte der Beschäftigten täglich getestet (allerdings enthält die Stichprobe nur zehn Betriebe). Ein weiterer hoher Anteil findet

Tabelle 3
**Tests pro Kopf und Tag im Dezember 2021**

| | Durchschnitt (ungewichtet, %) | Durchschnitt (gewichtet, %)[2] | Median (%) | N Betriebe | N Beschäftigte |
|---|---|---|---|---|---|
| **Nach Betriebsgrößen** | | | | | |
| Kleinstunternehmen | 27,3 | 24,3 | 14,3 | 167 | 926 |
| Kleinunternehmen | 23,8 | 22,7 | 15,8 | 265 | 6.950 |
| Mittlere Unternehmen | 21,3 | 20,5 | 13,3 | 189 | 22.515 |
| Großbetriebe | 12,9 | 10,5 | 10,0 | 56 | 48.541 |
| **Nach ausgewählten Branchen** | | | | | |
| Baugewerbe | 25,5 | 19,5 | 20,0 | 143 | 15.389 |
| Dienstleistungen[3] | 22,1 | 8,8 | 12,5 | 156 | 13.284 |
| Energieversorgung | 21,4 | 7,0 | 10,9 | 12 | 13.812 |
| Gastgewerbe | 64,5 | 53,4 | 29,2 | 10 | 427 |
| Gesundheits- und Sozialwesen | 39,0 | 31,5 | 40,0 | 35 | 6.777 |
| Handel | 16,2 | 11,1 | 11,8 | 35 | 1.784 |
| Information und Kommunikation | 16,8 | 12,7 | 8,3 | 25 | 2.219 |
| Verarbeitendes Gewerbe | 19,6 | 14,4 | 13,4 | 84 | 12.742 |
| Verkehr und Lagerei | 18,8 | 13,4 | 8,9 | 47 | 4.404 |

[1] Frage: „Wie hoch ist die Anzahl der durchschnittlich durchgeführten COVID-19 Antigen-Schnelltests am Tag?“, Antworten im Verhältnis zur Beschäftigtenzahl im Betrieb. [2] nach Zahl der Beschäftigten. [3] Siehe Fußnote 2 Tabelle 1.

sich im Gesundheits- und Sozialwesen. Beide Bereiche stehen aufgrund der Vielzahl sozialer Kontakte im besonderen Brennpunkt möglicher Verbreitungswege von COVID-19 und haben zudem geringere Möglichkeiten für mobiles Arbeiten als andere Branchen. Dass mobiles Arbeiten die Belastung durch erforderliche Tests reduzieren kann, zeigen die vergleichsweise niedrigen Raten in Branchen mit hohen Homeoffice-Anteilen, wie z. B. Dienstleistungen, Energieversorgung, Information und Kommunikation.

## Mobiles Arbeiten: Homeoffice bzw. Remote Work

Mit den zunehmenden Inzidenzzahlen seit Ende September 2021 wurde auch die stärkere Nutzung von mobilem Arbeiten bzw. Homeoffice zunächst empfohlen und durch das aktuelle Infektionsschutzgesetz und der Homeoffice-Pflicht, wo möglich, konkretisiert. Mobiles Arbeiten ist in Unternehmen in unterschiedlicher Weise, z. B. in Abhän-

Tabelle 4

**Durchschnittlicher Anteil der Arbeitszeit im Homeoffice im Dezember 2021**

| | Durchschnitt (ungewichtet, %) | Durchschnitt (gewichtet, %)[2] | Median (%) | N Betriebe | N Beschäftigte |
|---|---|---|---|---|---|
| Nach Betriebsgrößen | | | | | |
| Kleinstunternehmen | 19,0 | 14,5 | 0,0 | 174 | 964 |
| Kleinunternehmen | 15,5 | 15,6 | 2,0 | 265 | 6.895 |
| Mittlere Unternehmen | 17,5 | 17,5 | 5,0 | 189 | 22.235 |
| Großbetriebe | 31,0 | 43,8 | 20,0 | 55 | 48.821 |
| Nach ausgewählten Branchen | | | | | |
| Baugewerbe | 9,3 | 15,0 | 0,3 | 140 | 14.486 |
| Dienstleistungen[3] | 30,9 | 50,2 | 20,0 | 157 | 15.085 |
| Energieversorgung | 38,8 | 67,9 | 35,0 | 12 | 13.812 |
| Gastgewerbe | 7,1 | 20,8 | 0,0 | 10 | 427 |
| Gesundheits- und Sozialwesen | 7,9 | 7,5 | 0,6 | 36 | 6.790 |
| Handel | 3,3 | 4,6 | 0,0 | 37 | 1.815 |
| Information und Kommunikation | 59,2 | 66,0 | 70,0 | 25 | 2.219 |
| Verarbeitendes Gewerbe | 12,7 | 15,9 | 5,0 | 86 | 12.990 |
| Verkehr und Lagerei | 11,7 | 10,8 | 0,0 | 47 | 4.369 |

[1] Frage: „Welchen Anteil der Arbeitszeit verbringen die Beschäftigten aktuell durchschnittlich im mobilen Arbeiten (Home-Office/Remote Work)?" [2] nach Zahl der Beschäftigten. [3] Siehe Fußnote 2 Tabelle 1.

Tabelle 5

**Beschäftigtenanteil im mobilen Arbeiten[1] im Dezember 2021**

| | Durchschnitt (ungewichtet, %) | Durchschnitt (gewichtet, %)[2] | Median (%) | N Betriebe | N Beschäftigte |
|---|---|---|---|---|---|
| Nach Betriebsgrößen | | | | | |
| Kleinstunternehmen | 14,6 | 10,0 | 0,0 | 173 | 962 |
| Kleinunternehmen | 8,4 | 8,0 | 0,0 | 260 | 6.761 |
| Mittlere Unternehmen | 8,6 | 8,3 | 1,0 | 189 | 22.215 |
| Großbetriebe | 21,1 | 36,9 | 10,0 | 56 | 50.631 |
| Nach ausgewählten Branchen | | | | | |
| Baugewerbe | 6,9 | 9,3 | 0,0 | 138 | 14.179 |
| Dienstleistungen[3] | 21,7 | 42,3 | 4,0 | 158 | 16.334 |
| Energieversorgung | 15,3 | 63,5 | 6,5 | 12 | 13.812 |
| Gastgewerbe | 6,7 | 21,5 | 0,0 | 9 | 377 |
| Gesundheits- und Sozialwesen | 1,1 | 1,0 | 0,0 | 36 | 6.790 |
| Handel | 0,7 | 0,9 | 0,0 | 37 | 1.815 |
| Information und Kommunikation | 33,8 | 41,8 | 20,0 | 25 | 2.219 |
| Verarbeitendes Gewerbe | 7,0 | 11,0 | 1,0 | 81 | 12.874 |
| Verkehr und Lagerei | 9,5 | 6,4 | 0,0 | 48 | 4.413 |

[1] Frage: „Welcher Anteil der Beschäftigten arbeitet aktuell permanent mobil bzw. von zuhause?" [2] nach Zahl der Beschäftigten. [3] Siehe Fußnote 2 Tabelle 1.

gigkeit von Wirtschaftszweig, Produktionsmethode, Art der Dienstleistung, möglich.

In Tabelle 4 sind Ergebnisse der Einschätzung zum durchschnittlichen Anteil der Arbeitszeit im Homeoffice dargestellt. Während dieser Anteil im Mittel der Betriebe bei 18,2 % lag, ist der Anteil beschäftigungsgewichtet mit 33,6 % deutlich größer – hierin spiegeln sich Größen- und Branchenunterschiede wider, die Auswirkungen auf die Möglichkeiten und Erfordernisse von Homeoffice haben. Dies bestätigt auch der Vergleich mit dem Median: In der Hälfte der Betriebe lag der Anteil bei 4 % oder darunter, in der anderen Hälfte entsprechend höher.

Speziell in Großbetrieben wurde ein großer Anteil der Arbeitszeit zuhause erbracht. Der durchschnittliche Anteil lag bei 31 %, beschäftigungsgewichtet sogar bei 43,8 %. Außerdem lassen sich klare sektorale Unterschiede erkennen.

In Branchen, wo das Homeoffice aus den Erfahrungen der vergangenen Jahre – und vermutlich seit Beginn der Pandemie – leicht und relativ friktionsfrei umsetzbar ist, arbeiteten große Teile der Beschäftigten im Dezember 2021 zeitweilig zuhause. Besonders hoch waren die Anteile in den Branchen Dienstleistungen, Energieversorgung und Information und Kommunikation; hier arbeiteten die Beschäftigten die Hälfte oder mehr ihrer Arbeitszeit von zuhause aus.

Tabelle 5 ergänzt die Angaben um den Anteil der Beschäftigten, die im Dezember zu keiner Zeit im Unternehmen arbeiteten. Sie hatten daher auch keine Arbeitswege zurückzulegen, was ein geringeres Ansteckungs- sowie Verbreitungsrisiko impliziert. Der Anteil im Durchschnitt der Betriebe lag zwar nur bei etwa 11,1 %, was aber mehr als einem Viertel der Beschäftigten entspricht (26,3 %). Der Anteil der permanent mobil arbeitenden Beschäftigten lag damit in der Bandbreite von 22 % bis 29 %, die auch infas für den Zeitraum der Homeoffice-Pflicht von Januar bis Mai 2021 ermittelt hatte (Corona Datenplattform, 2021). Ein solch restriktives Arbeitsmodell ist aus verschiedenen Gründen, z.B. aufgrund professioneller und sozialer Isolation oder der Organisation der Arbeitsabläufe, für die Mehrheit der Unternehmen nicht möglich oder nicht gewollt. Dies zeigt auch der Median: 50 % der Unternehmen beschäftigten keine Mitarbeiter:innen dauerhaft mobil.

Grundsätzlich sind die Muster des permanenten mobilen Arbeitens mit denen des anteiligen mobilen Arbeitens vergleichbar, die Anteile aber erwartungsgemäß etwas geringer. Insbesondere in Großbetrieben (36,9 %) und in den Branchen Dienstleistungen (42,3 %), Energieversorgung (63,5 %) und Information und Kommunikation (41,8 %) arbeiteten substanzielle Beschäftigtenanteile permanent zuhause und hatten somit keine arbeitsbedingten persönlichen Kontakte.

## Fazit

Durch die Änderung des Infektionsschutzgesetzes im November 2021 wurde die Abbildung der 3G- und 2G-Quoten auf betrieblicher Ebene knapp ein Jahr nach Beginn der Impfungen möglich. Die vorgestellte Analyse vermittelt erstmalig einen Eindruck über die Situation am Arbeitsplatz in niedersächsischen Betrieben auf Grundlage einer Kurzumfrage aus der zweiten Dezemberwoche. Im Mittel aller befragten Betriebe lag die 2G-Quote bei über 89 %. Die Auswertung macht zugleich Unterschiede in den 2G-Quoten zwischen den kleinsten, kleinen und mittleren Betrieben auf der einen Seite und den Großbetrieben auf der anderen Seite deutlich. Unterschiede zwischen den Branchen sind weniger stark ausgeprägt. Die Unternehmen nutzten Homeoffice im Dezember 2021 wieder in großem Umfang. Bereits bei Kleinstunternehmen ist jede/r zehnte Beschäftigte dauerhaft im Homeoffice, bei Großunternehmen mehr als jede/r Dritte. In einigen Branchen sind die Anteile mit mehr als 4 von 10 bzw. sogar mehr als 6 von 10 permanent im Homeoffice noch deutlich höher. Testraten und -bedarfe waren und sind sehr unterschiedlich zwischen den Branchen. Testerfordernisse und Homeoffice sind zudem teilweise komplementäre Maßnahmen: Unternehmen mit hoher Präsenzerfordernis und vielen Sozialkontakten müssen testen, während in Unternehmen ohne diese Anforderungen die Arbeit nach Hause verlagert wird. Im ersten Fall liegen die direkten Kosten vor allem auf der Arbeitgeberseite (Beschaffung der Tests, Verlust von Produktionskapazität durch verringerte Arbeitszeiten), im Fall von Homeoffice tragen dagegen vor allem die Beschäftigten die Kosten.

Inwieweit die hohen 2G-Quoten und Homeoffice-Anteile förderlich für die vergleichsweise niedrige Inzidenz und die geringe Zahl von Hospitalisierungen (Stand: Dezember 2021) in Niedersachsen im Bundesvergleich waren, kann mit den Daten nicht exakt quantifiziert werden. Ein positiver Beitrag lässt sich aber vermuten, da die betriebliche Umgebung einen wichtigen Teil der täglichen Kontakte und damit der Infektionsmöglichkeiten repräsentiert. Die ermittelten Werte können zudem als positives Indiz einer großen Akzeptanz der Impfung gewertet werden. Aufgrund der hohen Impfquoten ist von einer erfolgreichen Durchführung der aktuellen Boosterimpfungen auszugehen. Die Heterogenität in den Ergebnissen sollte bei der Entwicklung von Maßnahmen und politischen Entscheidungen stärker berücksichtigt werden. Anstelle von „One size fits all"-Maßnahmen sollte möglichst differenziert vorgegangen werden, um Vertrauen und Zustimmung zu erhalten und effizient zu agieren. Evidenzbasierte Politik sollte sich auf belastbare Daten stützen, Eingriffe und Maßnahmen sollten ausgewogen sein. Sie sollten neben der Wirksamkeit auch immer das Kosten-Nutzen-Verhältnis (Effizienz) berücksichtigen. Eine vergleichbare Erhebung könnte regelmäßig und am besten repräsentativ erfolgen, um die notwendigen Grundlagen für etwaige Eingriffe zu erstellen.
